# Supplementary material for: The 3D nuclear position and compartmentalization of genes prime their response to mechano-confinement
Source: bioRxiv. 2025 Nov 7:2025.11.06.686901. Preprint. [Version 1] doi: 10.1101/2025.11.06.686901 (PMC12637494; doi:10.1101/2025.11.06.686901)
Supplement: Supplement 1 [file media-1.pdf]

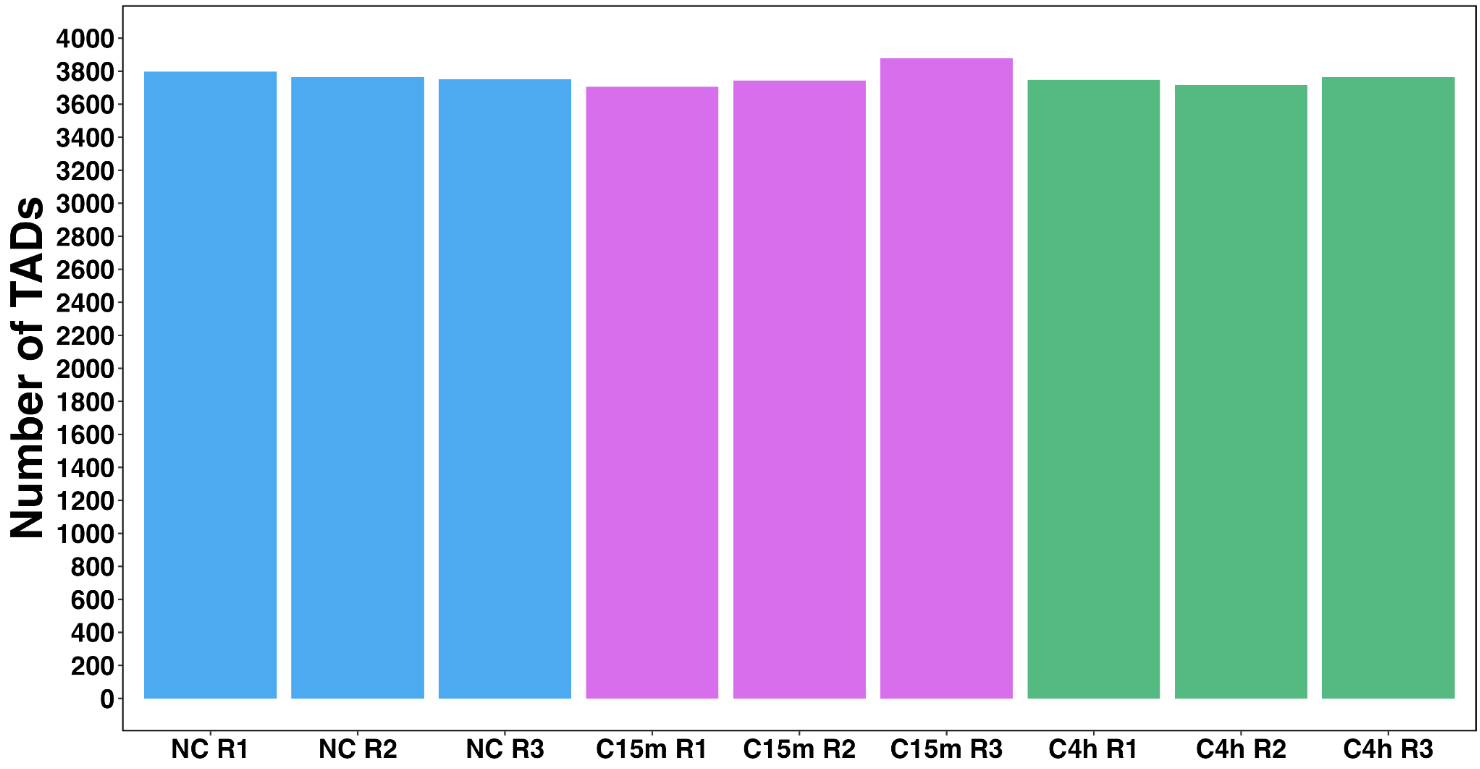

Fig. S1: The number of identified TADs in each replicate at 50kbp.

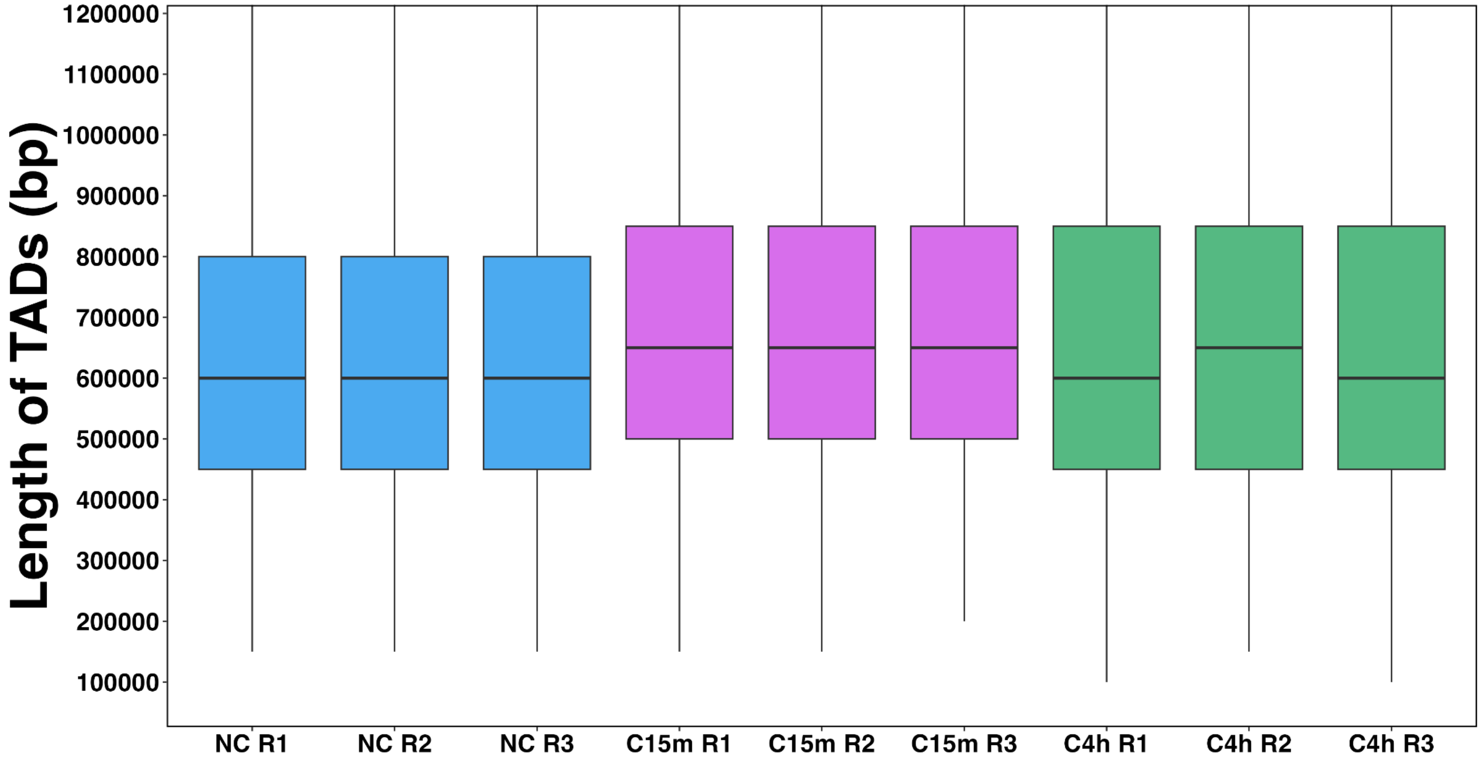

Fig. S2: Boxplot of the length of the identified TADs in each replicate at 50kbp.

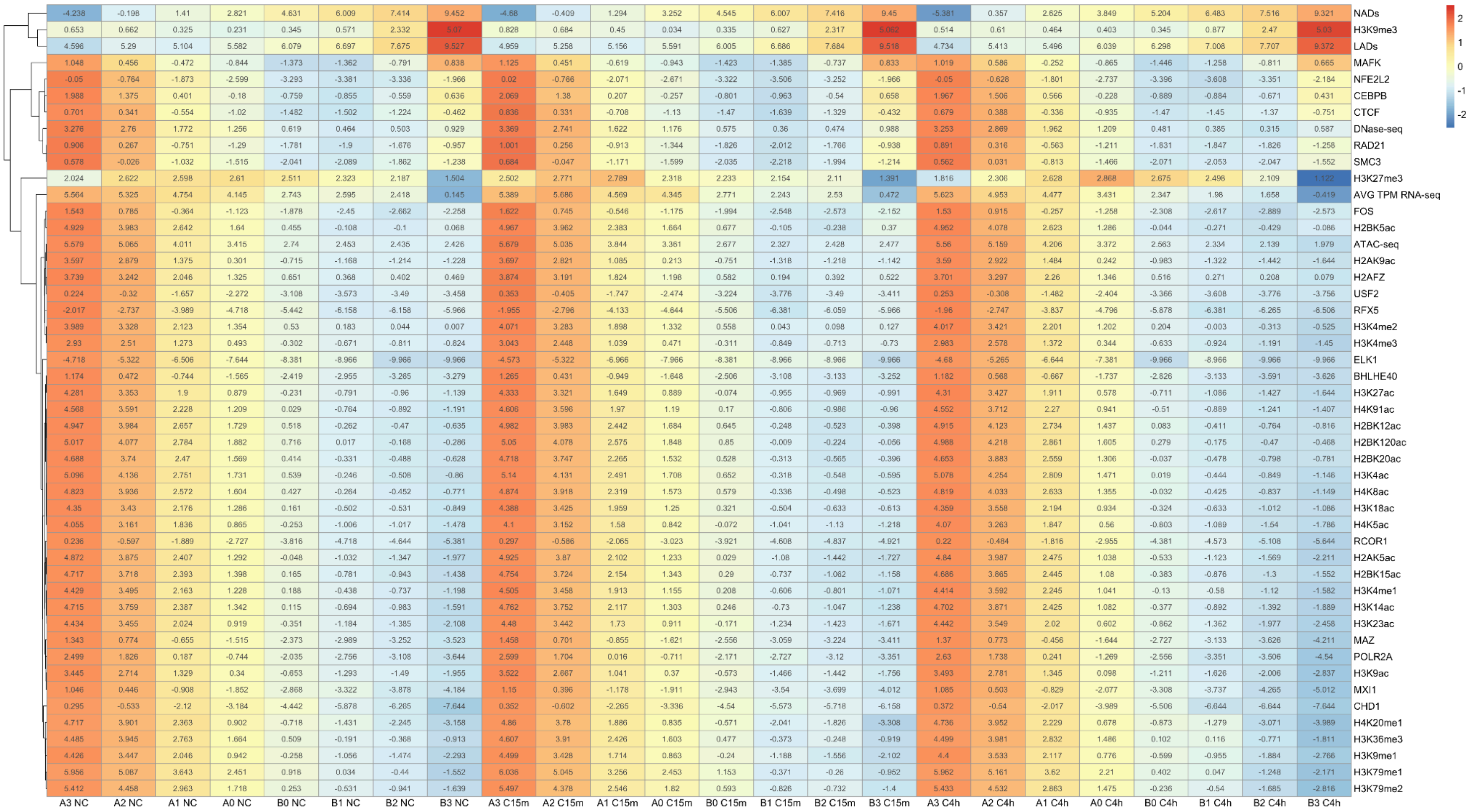

Fig. S3: Sub-compartment overlap with transcription factor and histone marker ChIP-seq data

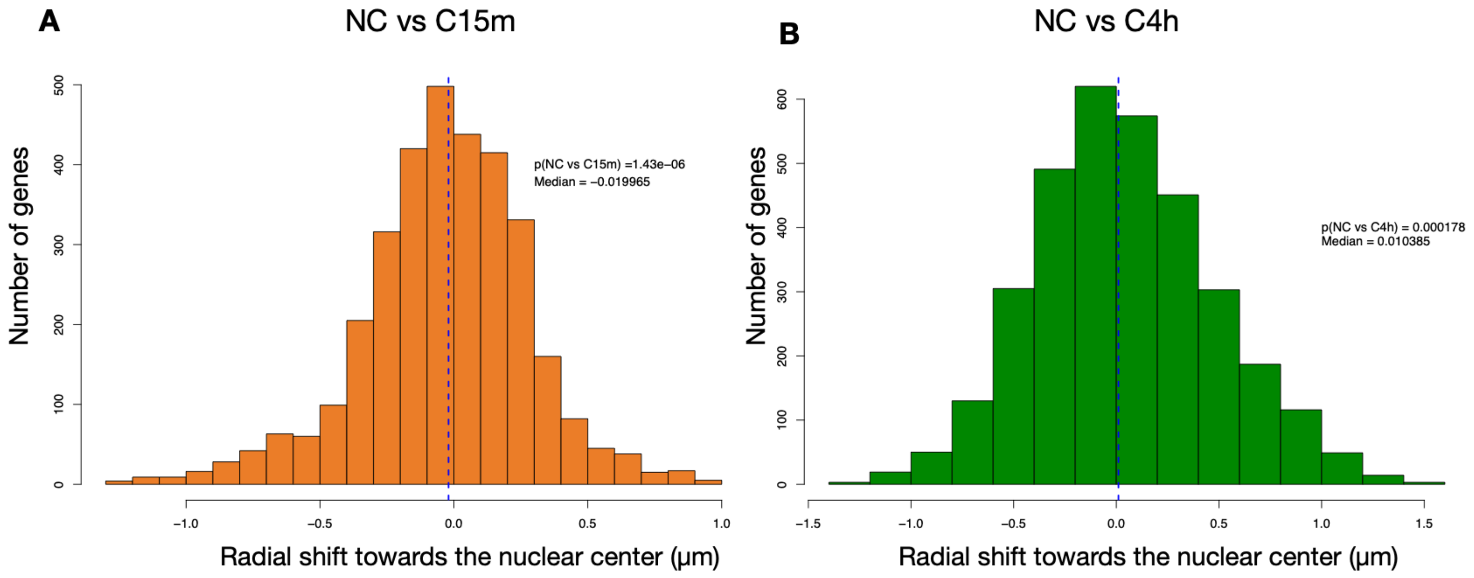

Fig. S4: Histogram showing the differences in median distances from the nuclear center for each TEC bead between NC vs. C15m (A) and NC vs. C4h (B) Chrom3D models. Negative and positive values indicate movement toward or away from the nuclear center, respectively. Pairwise Wilcoxon rank-sum tests were used to assess statistical significance of the differences in median distances between conditions.

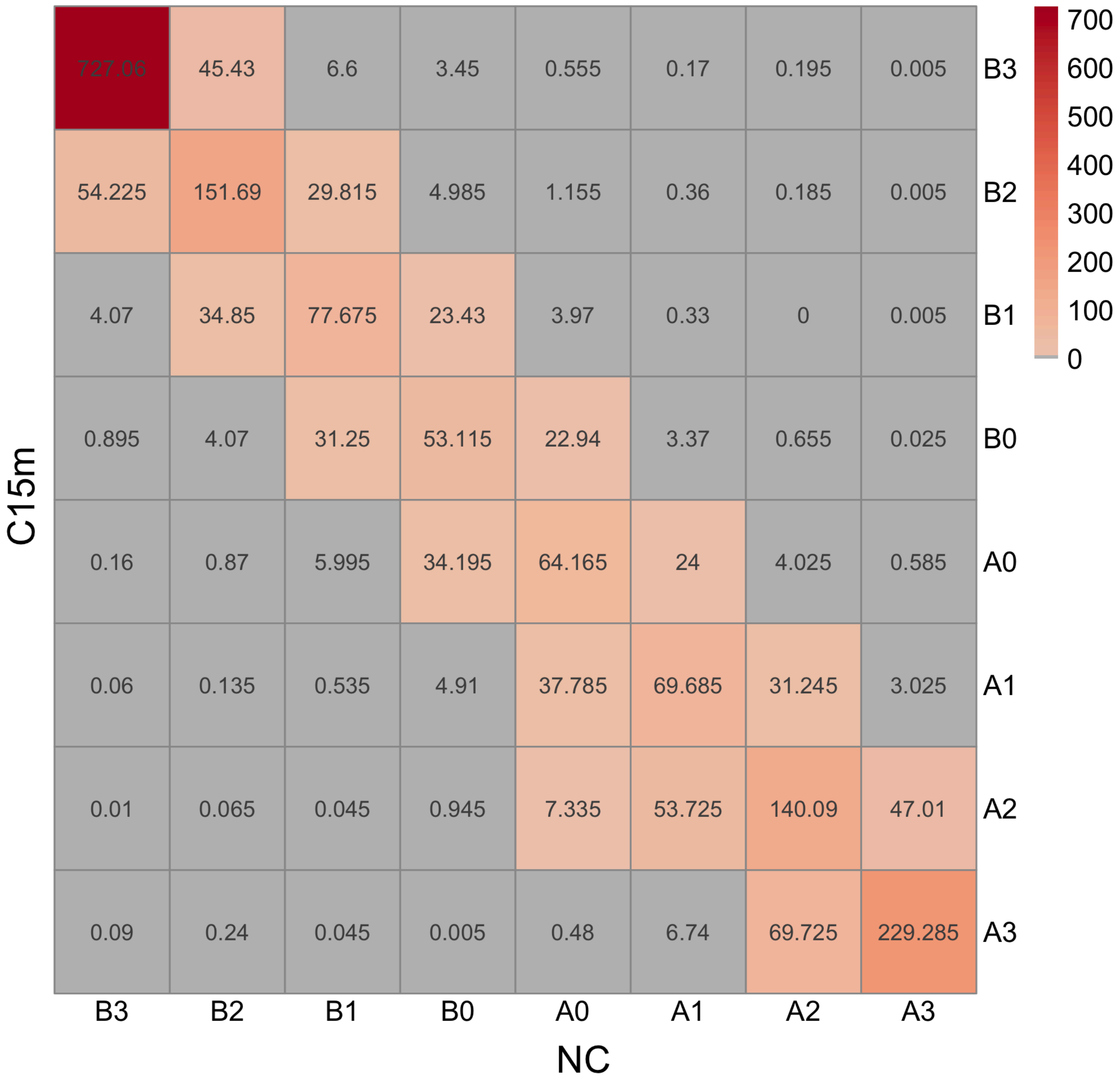

Fig. S5: Sub-compartment switches in the whole genome in Mbp NC vc. C15m

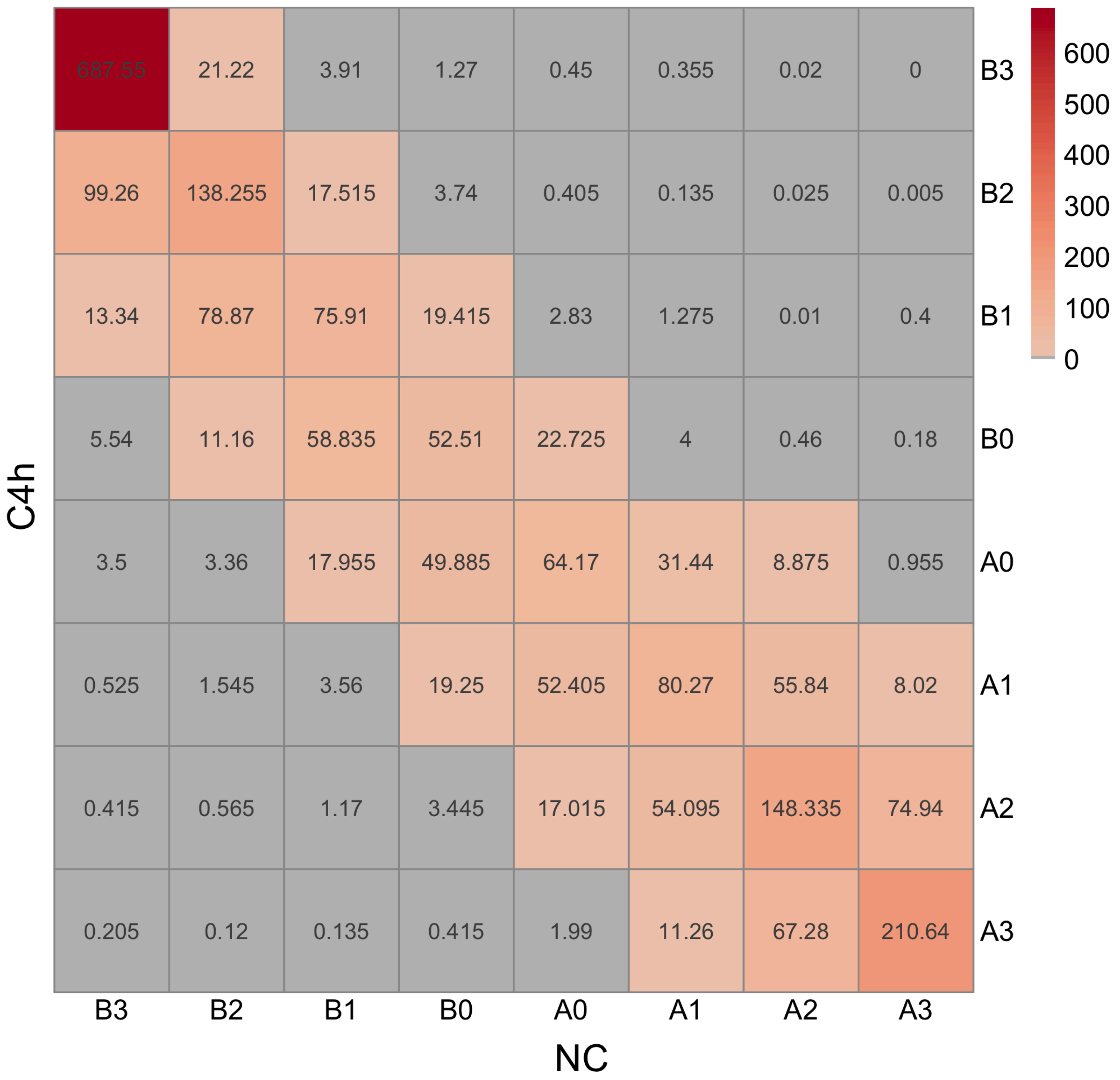

Fig. S6: Sub-compartment switches in the whole genome in Mbp NC vc. C4h

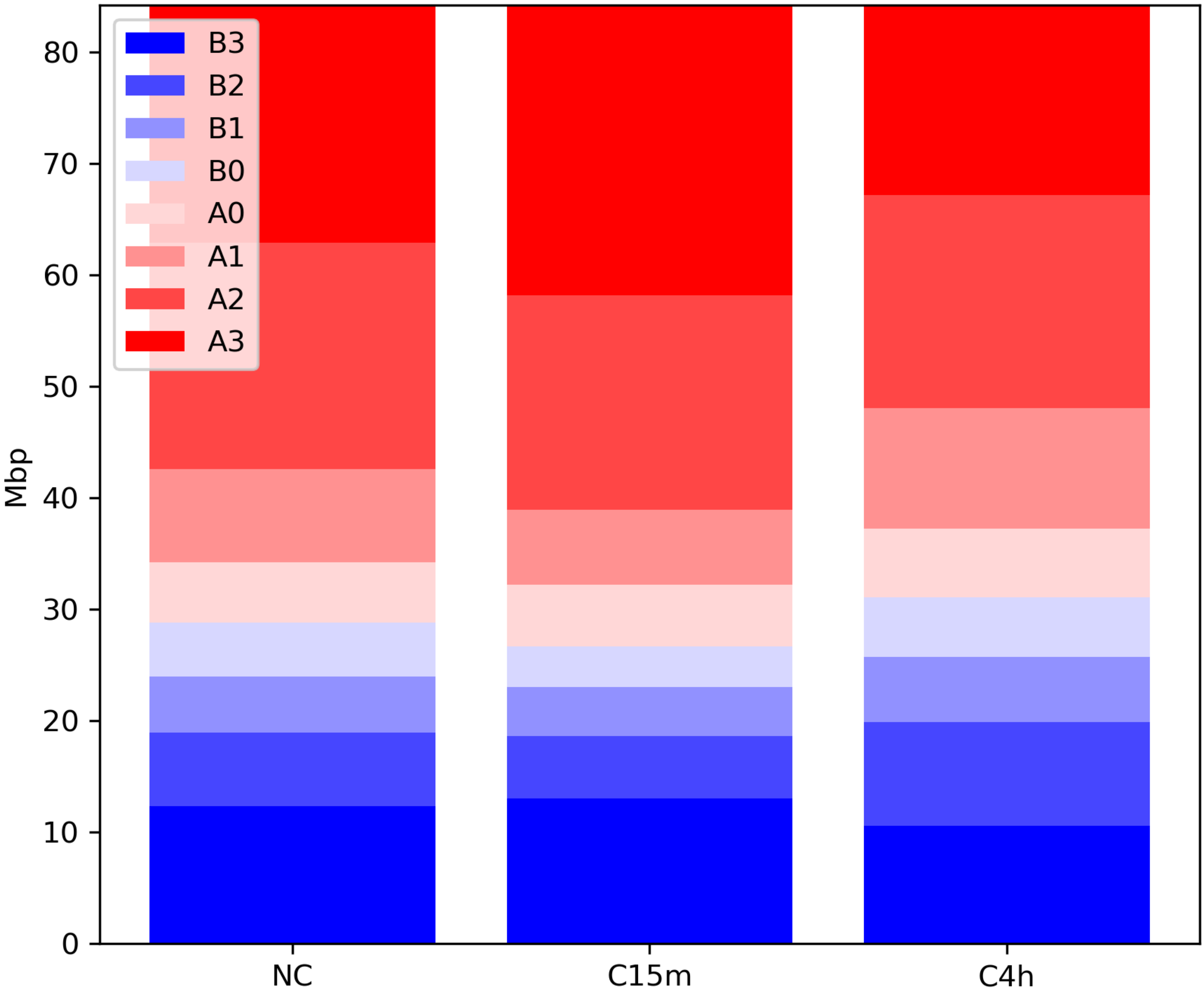

Fig. S7: Sub-compartment share in significant chromosome 9 inter-chromosomal interactions in NC vs. C15m

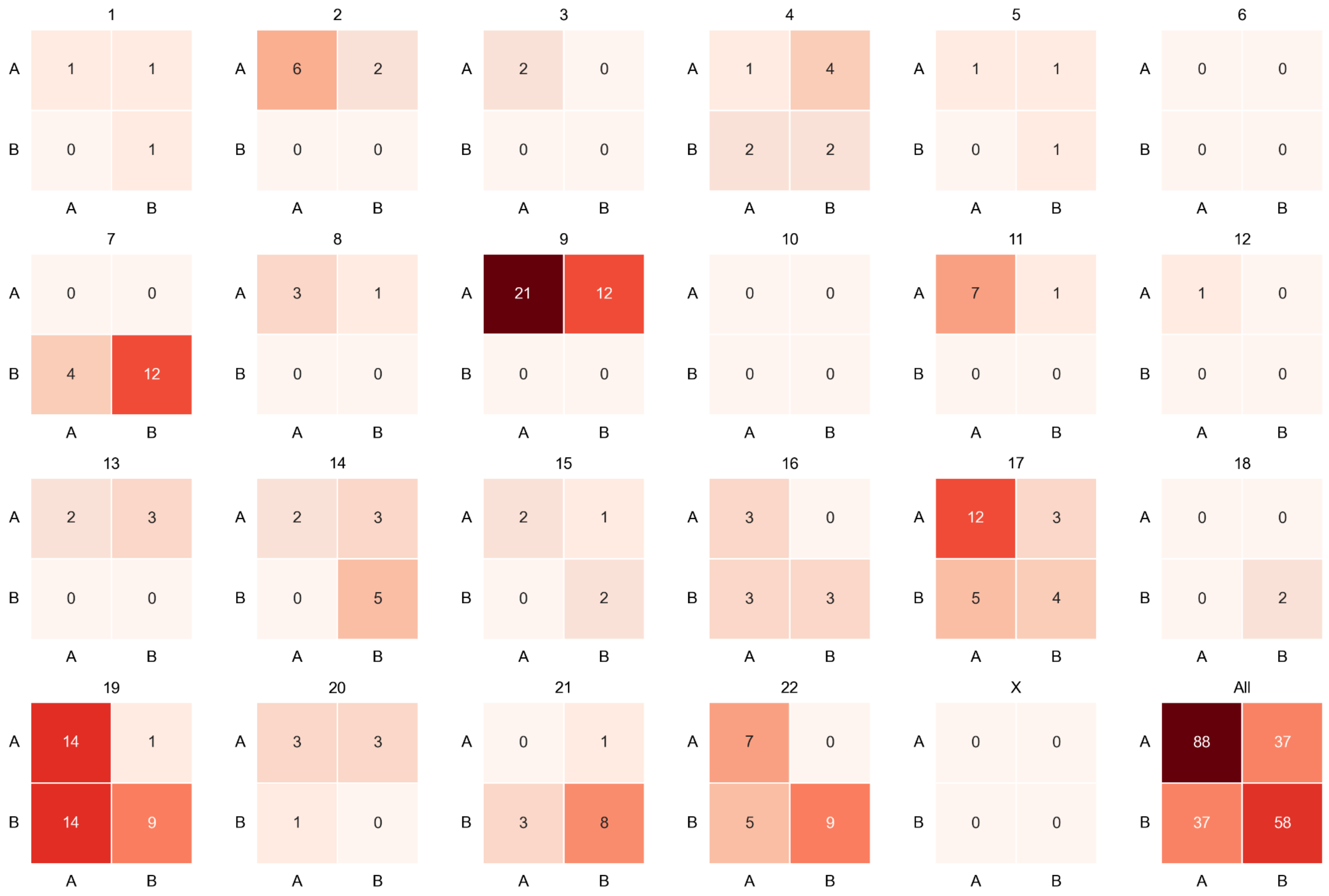

Fig. S8: Compartment status (A or B) for differentially interacting inter-chromosomal Hi-C regions in NC vs. C15m from each chromosome (indicated on top of each matrix), or jointly for all chromosomes (“All”).

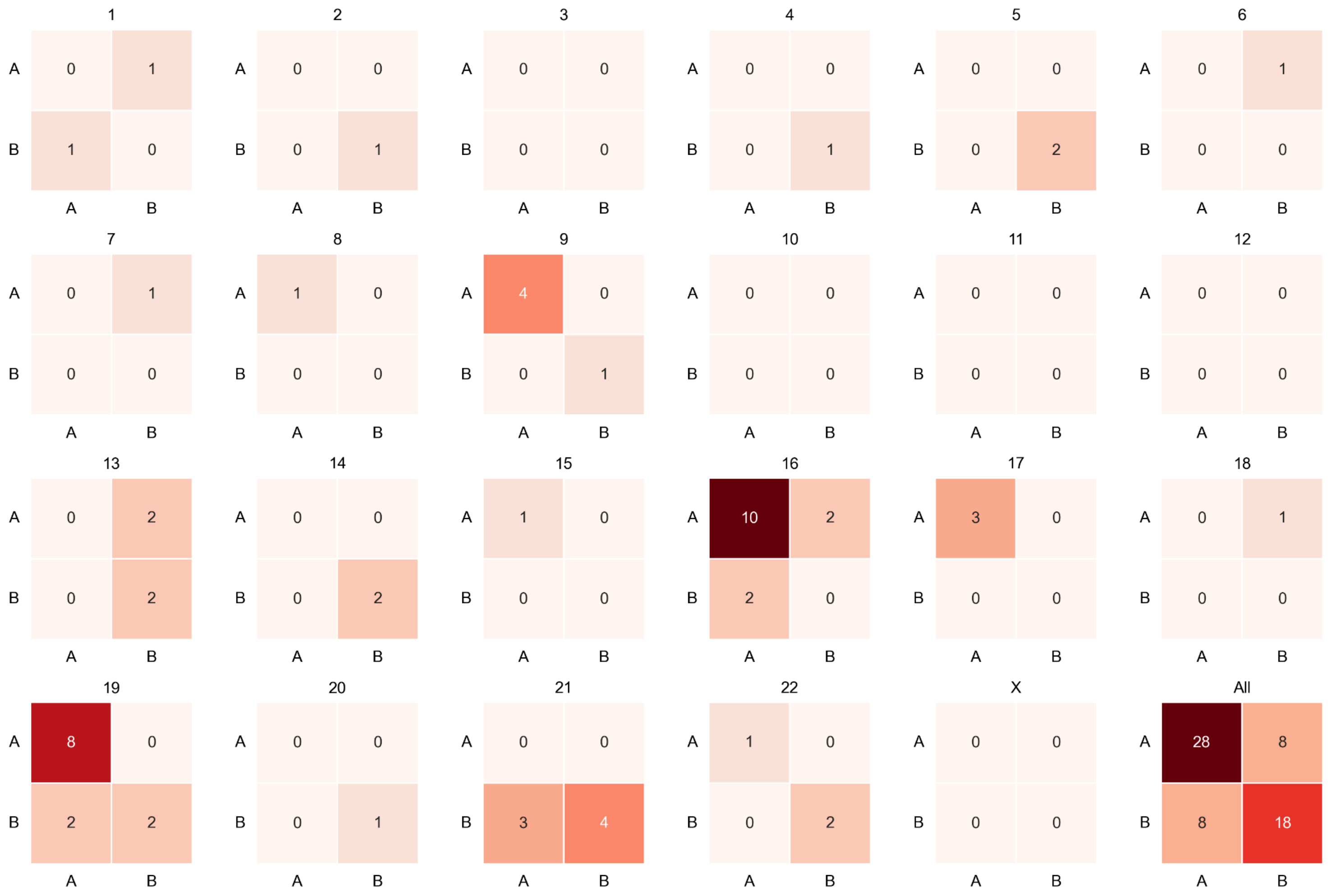

Fig. S9: Compartment status (A or B) for differentially interacting inter-chromosomal Hi-C regions in NC vs. C4h from each chromosome (indicated on top of each matrix), or jointly for all chromosomes (“All”).

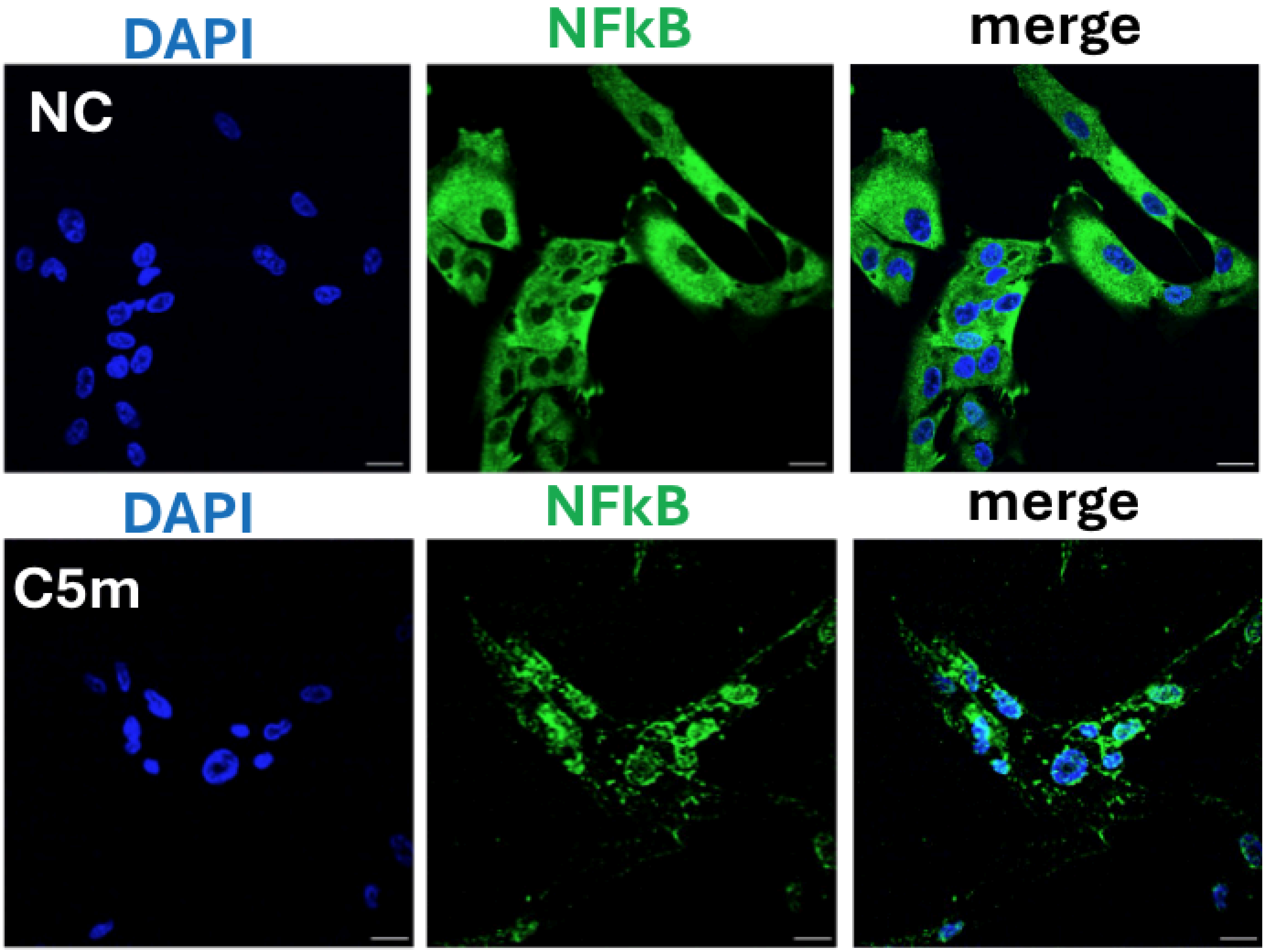

Fig. S10: Immunostaining of NFkB (green), and DAPI (blue) in NC (top) and cells confined for 5 minutes (C5m; bottom). Merge shown in the right panels.

A

	1	2	3	4	5	6	7	8
A	NT-4	IGFBP-3	FGF-4	Oncostatin M	MIP-1-delta	IL12-p40	I-309	Pos
B	Osteopontin	IGFBP-4	FGF-6	TPO	RANTES	IL-13	IL-1alpha	Pos
C	Osteoprotegerin	IL-16	FGF-7	VEGF	SCF	IL-15	IL-1beta	Pos
D	PARC	IP-10	FGF-9	PDGF-BB	SDF-1	IFN-gamma	IL-2	Pos
E	PIGF	LIF	Flt-3 Ligand	Leptin	TARC	MCP-1	IL-3	Neg
F	TGF- b 2	LIGHT	Fractalkine	BDNF	TGF-beta 1	MCP-2	IL-4	Neg
G	TGF- b 3	MCP-4	GCP-2	BLC	TNF-alpha	MCP-3	IL-5	ENA-78
H	TIMP-1	MIF	GDNF	CK beta 8-1	TNF-beta	M-CSF	IL-6	G-CSF
I	TIMP-2	MIP-3-alpha	HGF	Eotaxin	EGF	MDC	IL-7	GM-CSF
J	Pos	NAP-2	IGFBP-1	Eotaxin-2	IGF-1	MIG	IL-8	GRO
K	Pos	NT-3	IGFBP-2	Eotaxin-3	Angiogenin	MIP-1 beta	IL-10	GRO-alpha

B

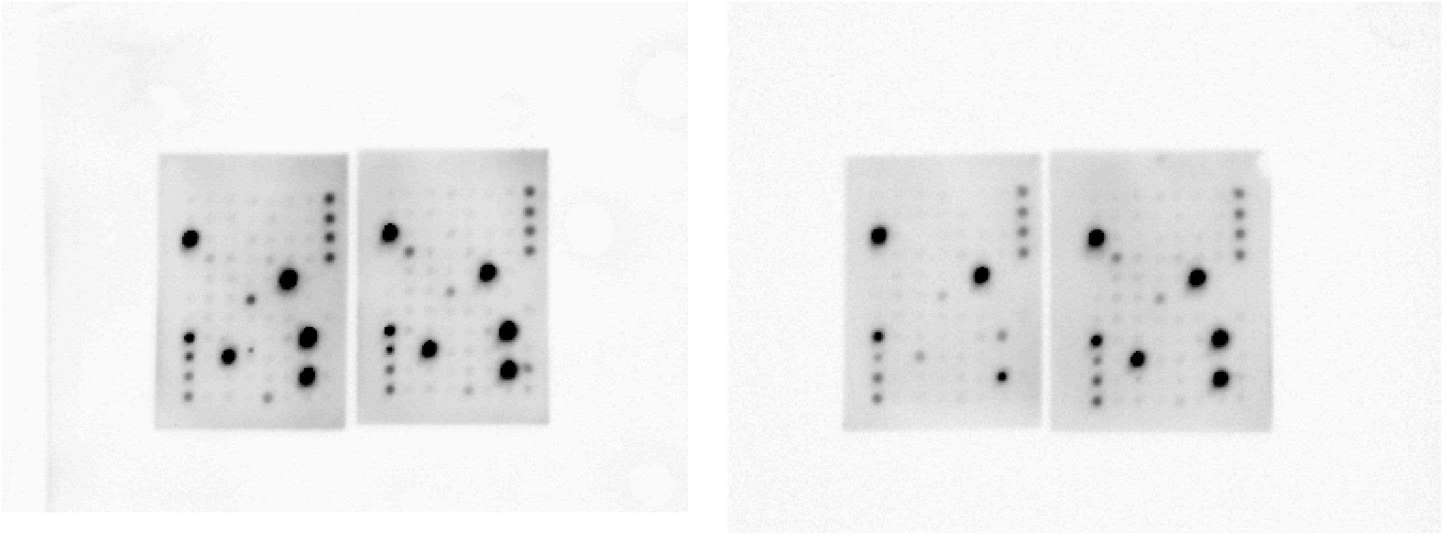

Fig. S11: A: Target map showing the assayed cytokines on the utilized C5 cytokine antibody array (RayBiotech, AAH-CYT-5) **B:** Images showing both replicates (left and right image), with each showing non-confined (NC) to the left, and confined at 4h (C4h) to the right.

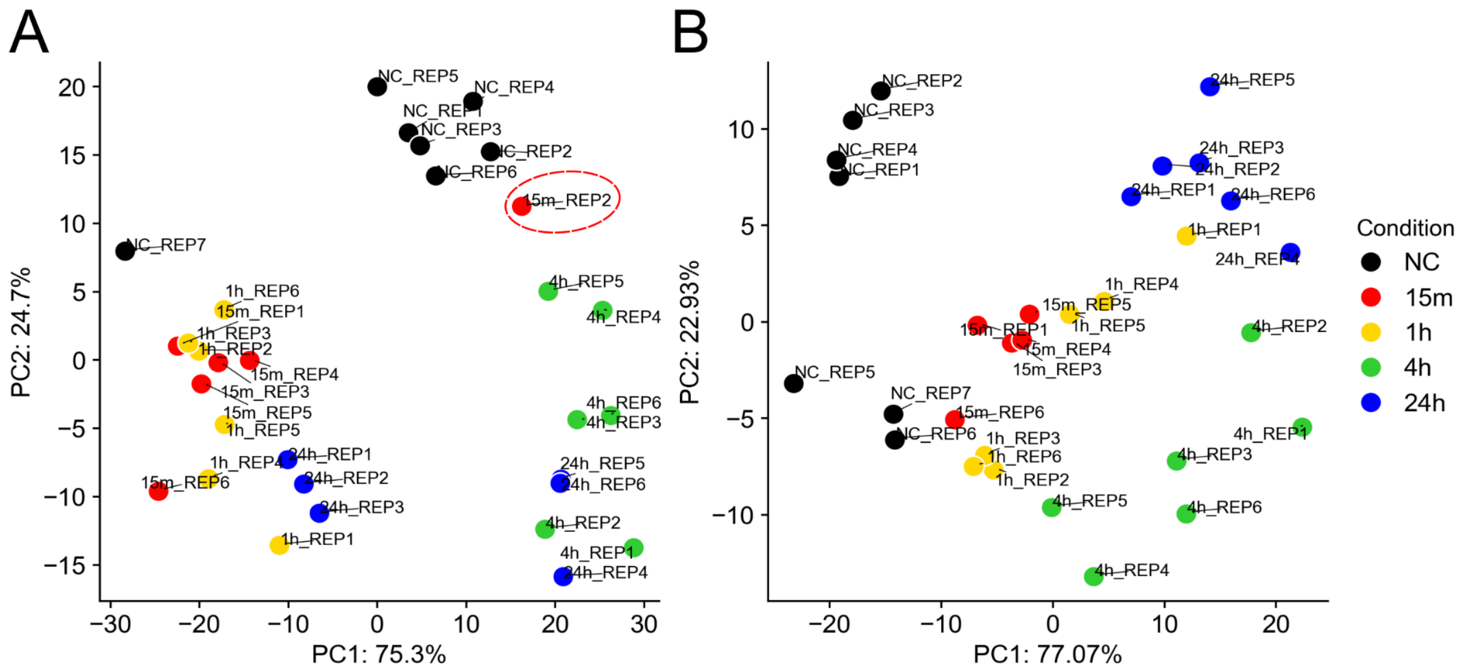

Fig. S12: Principal component analysis (PCA) of RNA-seq samples based on gene expression profiles. **A:** PCA plot of all sample replicates for each condition (shown in separate colors [see legend on the right]). The sample 15m_REP2 is highlighted by a red dotted circle. **B:** PCA plot after excluding sample 15m_REP2.
