# Supplementary material for: The 3D nuclear position and compartmentalization of genes prime their response to mechano-confinement
Source: bioRxiv. 2025 Nov 7:2025.11.06.686901. Preprint. [Version 1] doi: 10.1101/2025.11.06.686901 (PMC12637494; doi:10.1101/2025.11.06.686901)
Supplement: Supplement 2 [file media-2.pdf]

Table S1: Sub-compartment switching composition by TEC NC vs. C15m

**TEC**	**0**		**1**		**2**		**3**		**4**		**5**		**6**		**7**	
**Total**	6042		5214		2560		2164		356		552		213		153	
**Non-switch**	2715	0.45	2390	0.46	1183	0.46	1032	0.48	172	0.48	244	0.44	89	0.42	73	0.48
**Chromatin opening**	1109	0.18	915	0.18	434	0.17	383	0.18	49	0.14	95	0.172	34	0.16	26	0.17
**Chromatin closing**	706	0.12	746	0.14	240	0.09	299	0.14	52	0.15	61	0.11	29	0.14	17	0.11
**Facultative heterochromatin gain**	215	0.036	255	0.05	37	0.01	66	0.030	23	0.06	9	0.0163	7	0.03	7	0.046
**Facultative heterochromatin loss**	252	0.04	254	0.05	65	0.03	77	0.035	13	0.037	18	0.033	6	0.028	7	0.046

Table S2: Sub-compartment switching composition by TEC NC vs. C4h

**TEC**	**0**		**1**		**2**		**3**		**4**		**5**		**6**		**7**	
**Total**	6042		5214		2560		2164		356		552		213		153	
**Non-switch**	2353	0.39	2067	0.4	1082	0.42	937	0.43	149	0.42	221	0.40	69	0.32	59	0.39
**Chromatin opening**	1352	0.22	1286	0.25	429	0.17	443	0.20	74	0.21	99	0.179	50	0.23	42	0.27
**Chromatin closing**	825	0.14	698	0.13	346	0.14	334	0.15	50	0.14	80	0.14	33	0.15	15	0.098
**Facultative heterochromatin gain**	337	0.06	317	0.06	80	0.031	89	0.041	23	0.065	24	0.043	9	0.04	11	0.072
**Facultative heterochromatin loss**	315	0.05	362	0.07	70	0.027	105	0.049	21	0.059	19	0.034	14	0.066	11	0.072

Table S3: Mean log2FC per cluster and condition, rounded to 3 decimal places

TEC	C15m	C1h	C4h	C24h
0	0.008	0.309	0.038	0.086
1	0.396	-0.398	-0.172	-0.25
2	-0.851	1.184	0.443	0.63
3	-0.206	0.393	-0.118	0.033
4	2.14	-2.16	-0.858	-1.73
5	-1.28	4.008	0.247	1.038
6	0.405	-0.434	1.504	1.423
7	-1.574	1.246	3.291	3.54

Table S4: Hi-C interaction statistics by replicate and condition

	NC_rep1	NC_rep2	NC_rep3	C15m_rep1	C15m_rep2	C15m_rep3	C4h_rep1	C4h_rep2	C4h_rep3
valid_interaction	481153485	566639409	610851332	554462543	599472699	697116600	555197933	702914209	547940609
valid_interaction_rmdup	402519041	477289162	497843748	386142107	365213766	454286864	459671694	581461162	456636315
trans_interaction	61881584	64460192	53305729	54082262	62963926	73608358	80671856	118613919	89725395
cis_interaction	340637457	412828970	444538019	332059845	302249840	380678506	378999838	462847243	366910920
cis_shortRange	131738738	156334274	180647614	131871725	107109512	172266877	141944933	145993135	143407393
cis_longRange	208898719	256494696	263890405	200188120	195140328	208411629	237054905	316854108	223503527
Valid_interaction_pairs_FF	112015871	132063886	139957461	123351464	137760899	145493640	126702723	165113372	123845440
Valid_interaction_pairs_RR	112092525	132109281	140096374	123443846	137882037	145596517	126726396	165245739	123888339
Valid_interaction_pairs_RF	109845362	129673457	136543804	121680370	136430673	140980091	123557171	163363331	120558892
Valid_interaction_pairs_FR	147199727	172792785	194253693	185986863	187399090	265046352	178211643	209191767	179647938
Dangling_end_pairs	3877003	2810313	5121418	1874453	2053604	2812663	4105637	4917308	7797807
Religation_pairs	7764531	6924555	11143466	5143712	5070752	7621754	8671069	9854691	13816127
Self_Cycle_pairs	4219	5860	5888	10222	14827	17347	6345	4382	4812
Single-end_pairs	0	0	0	0	0	0	0	0	0
Filtered_pairs	0	0	0	0	0	0	0	0	0
Dumped_pairs	1217	1177	1507	706	710	1034	1435	993	1527

Table S5: Sub-compartment share by condition and replicate in percentage

sub_com	NC_rep1	NC_rep2	NC_rep3	C15min_rep1	C15min_rep2	C15min_rep3	C4h_rep1	C4h_rep2	C4h_rep3
A3	11.29	9.72	10.69	10.79	10.98	11.8	10.08	11.5	10.94
A2	11.77	10.81	11.59	11.42	11.42	10.82	12.47	12.59	13.29
A1	9.45	8.76	9.06	8.67	8.35	7.49	10.77	10.62	11.42
A0	8.13	7.96	8.77	7.47	8.57	7.08	9.01	9.33	9.51
B0	7.46	8.45	8.03	7.22	7.33	6.22	8.34	7.47	7.65
B1	8.58	8.96	8.59	7.41	7.58	7.93	9.21	8.32	7.89
B2	10.32	10.42	10.7	9.6	9.49	12	10.28	9.52	10.14
B3	26.95	28.91	26.6	26.84	25.69	26.68	23.92	24.84	23.22
NA	6.04	6.01	5.99	10.58	10.61	9.96	5.93	5.81	5.94

Table S6: Sub-compartment share by condition and replicate in Mbp

sub_com	NC_rep1	NC_rep2	NC_rep3	C15min_rep1	C15min_rep2	C15min_rep3	C4h_rep1	C4h_rep2	C4h_rep3
A3	342.265	294.72	323.905	327.175	332.725	357.735	305.58	348.435	331.59
A2	356.675	327.75	351.19	346.235	346.05	327.92	377.905	381.665	402.815
A1	286.575	265.43	274.555	262.68	253.085	227.13	326.445	322.035	346.025
A0	246.41	241.315	265.775	226.31	259.665	214.61	273.045	282.78	288.23
B0	226.015	256.025	243.275	218.955	222.035	188.66	252.815	226.53	231.775
B1	260.15	271.515	260.24	224.615	229.82	240.505	279.095	252.215	239.275
B2	312.96	315.83	324.3	290.985	287.58	363.805	311.655	288.495	307.48
B3	816.935	876.365	806.31	813.6	778.64	808.84	724.905	752.805	703.805
NA	183.11	182.145	181.545	320.54	321.495	301.89	179.65	176.135	180.1

Table S7: Median distance from the nucleus center and the standard deviation in Chrom3D models per condition and subcompartment

subcompartment	median distance ± std - NC	median distance ± std - C15m	median distance ± std - C4h
A3	3.35 ± 0.33	3.24 ± 0.43	3.43 ± 0.37
A2	3.17 ± 0.33	3.04 ± 0.44	3.35 ± 0.33
A1	3.30 ± 0.32	3.33 ± 0.46	3.43 ± 0.33
A0	3.42 ± 0.32	3.60 ± 0.42	3.49 ± 0.36
B0	3.60 ± 0.32	3.45 ± 0.40	3.62 ± 0.34
B1	3.65 ± 0.32	3.64 ± 0.42	3.66 ± 0.32
B2	3.64 ± 0.30	3.66 ± 0.40	3.73 ± 0.35
B3	3.75 ± 0.29	3.62 ± 0.40	3.70 ± 0.31

Table S8: pairwise Wilcox results of the median subcompartment distance from the nucleus center per sub-compartment within NC Chrom3D simulations

	A3	A2	A1	A0	B0	B1	B2
A3							
A2	4.54e-03						
A1	1.00e+00	1.61e-02					
A0	6.02e-01	1.24e-05	2.73e-01				
B0	7.17e-08	8.23e-16	2.19e-09	9.71e-05			
B1	8.47e-09	1.10e-16	1.87e-10	8.53e-06	1.00e+00		
B2	8.86e-10	4.76e-18	2.23e-11	2.26e-06	1.00e+00	1.00e+00	
B3	7.12e-15	6.30e-23	6.54e-17	2.73e-11	5.00e-03	7.23e-02	8.82e-02

Table S9: pairwise Wilcox results of the median cluster distance from the nucleus center per sub-compartment within C15m Chrom3D simulations

	A3	A2	A1	A0	B0	B1	B2
A3							
A2	1.82e-04						
A1	4.93e-01	5.58e-07					
A0	1.61e-10	4.57e-16	4.08e-07				
B0	7.61e-06	2.76e-12	7.70e-03	2.39e-02			
B1	7.75e-12	5.83e-17	2.55e-08	1.00e+00	2.81e-03		
B2	3.38e-13	4.22e-18	4.81e-10	4.93e-01	6.71e-05	1.00e+00	
B3	3.95e-11	1.27e-16	1.45e-07	1.00e+00	1.66e-02	1.00e+00	4.93e-01

Table S10: pairwise Wilcox results of the median cluster distance from the nucleus center per sub-compartment within C4h Chrom3D simulations

	A3	A2	A1	A0	B0	B1	B2
A3							
A2	2.56e-01						
A1	1.00e+00	4.97e-01					
A0	1.00e+00	5.56e-03	5.87e-01				
B0	9.43e-05	7.63e-09	1.89e-05	7.07e-03			
B1	1.00e-07	9.67e-12	3.78e-08	2.72e-05	7.37e-01		
B2	1.06e-08	6.01e-13	1.98e-09	3.85e-06	2.56e-01	1.00e+00	
B3	1.20e-08	5.46e-13	1.76e-09	3.01e-06	2.08e-01	1.00e+00	1.00e+00

Table S11: Wilcoxon rank sum test results of the median subcompartment distance from the nucleus center in each Chrom3D simulation model per condition and subcompartment pair.

Condition 1	Condition 2	Subcomp.	P-value
NC	C15m	A3	0.509
NC	C15m	A2	0.185
NC	C15m	A1	0.172
NC	C15m	A0	2.20 × 10^-07
NC	C15m	B0	0.040
NC	C15m	B1	0.329
NC	C15m	B2	0.122
NC	C15m	B3	0.008
NC	C4h	A3	0.002
NC	C4h	A2	2.28 × 10^-06
NC	C4h	A1	5.89 × 10^-04
NC	C4h	A0	0.003
NC	C4h	B0	0.193
NC	C4h	B1	0.069
NC	C4h	B2	0.026
NC	C4h	B3	0.619
C15m	C4h	A3	3.82 × 10^-05
C15m	C4h	A2	5.04 × 10^-09
C15m	C4h	A1	0.043
C15m	C4h	A0	0.003
C15m	C4h	B0	6.22 × 10^-04
C15m	C4h	B1	0.392
C15m	C4h	B2	0.509
C15m	C4h	B3	0.011

Table S12: Median distance from the nucleus center and the standard deviation in Chrom3D models per condition and TEC

TEC	median distance ± std - NC	median distance ± std - C15m	median distance ± std - C4h
0	3.66 ± 0.32	3.62 ± 0.42	3.72 ± 0.36
1	3.71 ± 0.32	3.60 ± 0.44	3.70 ± 0.35
2	3.06 ± 0.31	2.96 ± 0.43	3.06 ± 0.33
3	3.37 ± 0.31	3.31 ± 0.41	3.44 ± 0.32
4	3.62 ± 0.32	3.54 ± 0.42	3.66 ± 0.34
5	3.16 ± 0.33	3.12 ± 0.43	3.21 ± 0.35
6	3.30 ± 0.31	3.25 ± 0.40	3.40 ± 0.34
7	3.48 ± 0.35	3.37 ± 0.45	3.48 ± 0.42

Table S13: pairwise Wilcox results of the median cluster distance from the nucleus center per cluster within NC Chrom3D simulations

	0	1	2	3	4	5	6
0							
1	3.82e-01						
2	6.55e-23	4.03e-24					
3	4.61e-09	1.01e-10	1.06e-10				
4	3.25e-01	1.50e-01	2.00e-20	6.89e-06			
5	1.72e-18	6.88e-20	1.47e-01	1.42e-05	1.74e-15		
6	8.62e-13	1.13e-14	7.35e-07	2.04e-01	1.43e-09	1.10e-02	
7	7.06e-04	3.72e-05	2.81e-14	1.50e-01	5.64e-02	4.61e-09	1.16e-03

Table S14: pairwise Wilcox results of the median cluster distance from the nucleus center per cluster within C15m Chrom3D simulations

	0	1	2	3	4	5	6
0							
1	7.42e-01						
2	8.75e-20	2.87e-20					
3	2.68e-08	3.38e-09	3.16e-10				
4	3.91e-01	3.01e-01	8.17e-18	3.31e-06			
5	3.03e-15	6.40e-16	9.96e-03	2.83e-05	3.24e-13		
6	1.75e-11	2.11e-12	2.54e-07	3.04e-01	5.09e-09	9.52e-03	
7	3.63e-05	9.29e-06	1.75e-11	4.46e-01	3.11e-03	1.63e-06	2.90e-02

Table S15: pairwise Wilcox results of the median cluster distance from the nucleus center per cluster within C4h Chrom3D simulations

	0	1	2	3	4	5	6
0							
1	9.39e-01						
2	3.93e-24	4.90e-24					
3	9.71e-10	7.34e-10	1.18e-13				
4	4.01e-01	4.01e-01	3.79e-22	1.50e-06			
5	8.70e-19	9.79e-19	3.82e-03	1.86e-07	1.18e-16		
6	1.46e-12	8.46e-13	3.12e-10	2.81e-01	1.32e-09	5.85e-04	
7	3.19e-05	1.85e-05	8.37e-16	4.01e-01	3.82e-03	4.91e-10	8.54e-03

Table S16: Wilcoxon rank sum test results of the median cluster distance from the nucleus center in each Chrom3D simulation model per condition and subcompartment pair.

Condition 1	Condition 2	TEC	P-value
NC	C15m	0	0.537
NC	C15m	1	0.273
NC	C15m	2	0.191
NC	C15m	3	0.517
NC	C15m	4	0.460
NC	C15m	5	0.776
NC	C15m	6	0.614
NC	C15m	7	0.227
NC	C4h	0	0.073
NC	C4h	1	0.392
NC	C4h	2	0.755
NC	C4h	3	0.031
NC	C4h	4	0.088
NC	C4h	5	0.151
NC	C4h	6	0.024
NC	C4h	7	0.177
C15m	C4h	0	0.014
C15m	C4h	1	0.035
C15m	C4h	2	0.095
C15m	C4h	3	0.003
C15m	C4h	4	0.011
C15m	C4h	5	0.063
C15m	C4h	6	0.007
C15m	C4h	7	0.007

Table S17: Fisher's exact test results of chromatin opening vs closing in upregulated clusters (TEC 2, 5, 6, 7) vs. downregulated TECs (TEC 1, 3, 4)

P-value = 3.805e-05; Odds ratio = 0.7234678

	Chromatin closing in C15m	Chromatin opening in C15m
Upregulated TECs	347	589
Downregulated TECs	1097	1347

P-value = 0.0008173; Odds ratio 1.27386

	Chromatin closing in C4h	Chromatin opening in C4h
Upregulated TECs	474	620
Downregulated TECs	1082	1803

Table S18: Cellular localization of p-NF-kB in IMR90 cells (n.o. cells).*

	Condition:	NC	C5m
Localization:	Cytoplasm	107	2
	Nucleus	3	139

* Fisher's exact test of [NC vs. C5m] vs. [Cytoplasm vs. Nucleus] gives P<2.2e-16

Table S19: Cellular localization of NF-kB in IMR90 cells (n.o. cells).*

	Condition:	NC	C5m
Localization:	Cytoplasm	123	3
	Nucleus	1	104

* Fisher's exact test of [NC vs. C5m] vs. [Cytoplasm vs. Nucleus] gives P<2.2e-16

Table S20: Intensity values from two replicates using the cytokine antibody array (RayBiotech, AAH-CYT-5).

Factor	Rep1 (intensity)	Rep2 (intensity)	Avg. (intensity)	S.D.
TIMP1	1.75416733	1.25902193	1.50659463	0.35012067
TIMP2	2.00292586	1.55761751	1.78027169	0.31488056
OPG	1.72867408	1.06321263	1.39594335	0.4705523
CXCL10	4.7924873	2.86982663	3.83115697	1.35952639
HGF	1.78113965	6.57749225	4.17931595	3.39153345
TGFb1	0.80829529	2.05545484	1.43187506	0.88187498
IL-3	1,51873155	1,0417141	1,28022282	0,33730228
IL-6	1,60492919	5,84406016	3,72449468	2,99751826
IL-8	1,62122753	2,2758561	1,94854182	0,4628923

Table S21: Fisher's exact test results of sub-compartment switches in NF-kappa target genes vs. non-NF-kappa target genes

Switch	Odds ratio	P-value
NC vs. C15m	1.33	0.022
NC vs. C4h	1.053	0.713
C15m vs. C4h	0.996	1
NC vs. C15m vs. C4h	1.145	0.299

Table S22: Publicly available data used for subcompartment overlap comparison analysis

Biosample	Assay	Target	Dataset accession	bed narrowPeak file accession
Homo sapiens IMR-90	Histone ChIP-seq	H3K9me3	ENCSR055ZZY	ENCFF098XMT
Homo sapiens IMR-90	Histone ChIP-seq	H3K27ac	ENCSR002YRE	ENCFF805GNH
Homo sapiens IMR-90	Histone ChIP-seq	H3K27me3	ENCSR431UUY	ENCFF336IXL
Homo sapiens IMR-90	Histone ChIP-seq	H3K4me3	ENCSR087PFU	ENCFF093NQC
Homo sapiens IMR-90	Histone ChIP-seq	H3K9ac	ENCSR219MYH	ENCFF309ZMM
Homo sapiens IMR-90	Histone ChIP-seq	H3K4me1	ENCSR831JSP	ENCFF611UWF
Homo sapiens IMR-90	TF ChIP-seq	CTCF	ENCSR000EFI	ENCFF203SRF
Homo sapiens IMR-90	ATAC-seq	DNA	ENCSR200OML	ENCFF243NTP
Homo sapiens IMR-90	ChIP-seq	Lamin B1	GSE36641	GSE36616
Homo sapiens IMR-90	Whole-Genome Tiling Array	Nucleolus-associated DNA	GSE78043	S1 table

Table S23: The total number of significant inter- and intra-chromosomal interactions in each sample identified by NCHG

Sample	Cis (FDR = 0.01; logRatio=2.0)	Trans (FDR = 0.01; logRatio=1.5)
NC rep1	23068	10014
NC rep2	16112	12307
NC rep3	14279	20179
NC consensus	16861	10411
C15m rep1	22858	20965
C15m rep2	23214	11167
C15m rep3	22907	59081
C15m consensus	21214	17681
C4h rep1	19159	11457
C4h rep2	4744	22767
C4h rep3	20096	8062
C4h consensus	13419	11110
